# Comparative sequence analyses reveal sites of ancestral chromosomal fusions in the Indian muntjac genome

**DOI:** 10.1186/gb-2008-9-10-r155

**Published:** 2008-10-28

**Authors:** Vicky Tsipouri, Mary G Schueler, Sufen Hu, NISC Comparative Sequencing Program, Amalia Dutra, Evgenia Pak, Harold Riethman, Eric D Green

**Affiliations:** 1 Genome Technology Branch, National Human Genome Research Institute, National Institutes of Health, 50 South Dr., Bethesda, Maryland, 20892, USA; 2 Molecular and Cellular Oncogenesis, Wistar Institute, 3601 Spruce Street, Philadelphia, Pennsylvania, 19104, USA; 3 NIH Intramural Sequencing Center (NISC), 5625 Fishers Ln., Rockville, Maryland, 20852, USA; 4 Genetic Disease Research Branch, National Human Genome Research Institute, National Institutes of Health, 49 Convent Dr., Bethesda, Maryland, 20892, USA

## Abstract

**Background:**

Indian muntjac (*Muntiacus muntjak vaginalis*) has an extreme mammalian karyotype, with only six and seven chromosomes in the female and male, respectively. Chinese muntjac (*Muntiacus reevesi*) has a more typical mammalian karyotype, with 46 chromosomes in both sexes. Despite this disparity, the two muntjac species are morphologically similar and can even interbreed to produce viable (albeit sterile) offspring. Previous studies have suggested that a series of telocentric chromosome fusion events involving telomeric and/or satellite repeats led to the extant Indian muntjac karyotype.

**Results:**

We used a comparative mapping and sequencing approach to characterize the sites of ancestral chromosomal fusions in the Indian muntjac genome. Specifically, we screened an Indian muntjac bacterial artificial-chromosome library with a telomere repeat-specific probe. Isolated clones found by fluorescence *in situ *hybridization to map to interstitial regions on Indian muntjac chromosomes were further characterized, with a subset then subjected to shotgun sequencing. Subsequently, we isolated and sequenced overlapping clones extending from the ends of some of these initial clones; we also generated orthologous sequence from isolated Chinese muntjac clones. The generated Indian muntjac sequence has been analyzed for the juxtaposition of telomeric and satellite repeats and for synteny relationships relative to other mammalian genomes, including the Chinese muntjac.

**Conclusions:**

The generated sequence data and comparative analyses provide a detailed genomic context for seven ancestral chromosome fusion sites in the Indian muntjac genome, which further supports the telocentric fusion model for the events leading to the unusual karyotypic differences among muntjac species.

## Abstract 2

Comparative mapping and sequencing was used to characterize the sites of ancestral chromosomal fusions in the Indian muntjac genome.

## Background

The number of chromosomes in the mammalian nuclear genome is generally well-confined, typically ranging from 36 to 60. There are, however, rare exceptions. At one extreme, the genome of the red viscacha rat (*Tympanoctomys barrerae*) consists of 102 chromosomes [1]; at the other extreme, that of the Indian muntjac (*Muntiacus muntjak vaginalis*) consists of a modest 6 and 7 chromosomes (in the female and male, respectively) [2]. Understanding the molecular basis for such radically different mammalian karyotypes would provide insight about the evolutionary history that has led to architecturally distinct genomes. Furthermore, comparative studies of mammalian karyotypes can more generally advance our understanding of vertebrate genome evolution [3].

Muntjacs belong to the suborder Ruminantia, which includes Moschidae (musk deer), Tragulidae (chevrotains and mouse deer), Antilocapridae (pronghorns), Giraffidae (giraffes and okapis), Bovidae (cattle, sheep, goats, and antelopes), and Cervidae (deer). The Cervidae family includes moose, caribou, deer, and muntjacs, with the various species inhabiting Europe, Asia, North Africa, and the Americas [4]. Muntjacs have interested cytogeneticists and genomicists because of the markedly different karyotypes that are present in closely related species [5]. In contrast to the strikingly low chromosome number in the Indian muntjac, its close relative - the Chinese muntjac (*Muntiacus reevesi*) - has a chromosome number that is more typical of a mammal, with 46 in both sexes [6]. The total genome size in Chinese and Indian muntjacs is believed to differ only slightly, with haploid C-values of 2.7 and 2.1 pg, respectively [7]; as such, the physical chromosome lengths vary tremendously between the species. Indian and Chinese Muntjacs are morphologically similar and can interbreed to produce viable (although sterile) offspring [8]. Interestingly, there are muntjac species with genomes with intermediate numbers of chromosomes: *Muntiacus feae *has 13 and 14 chromosomes in the female [9] and male [10], respectively, while both *Muntiacus crinifrons *and *Muntiacus gongshanensis *have 8 and 9 chromosomes in the female and male, respectively [11]. In general, muntjacs are thought to have been subjected to the fastest rate of evolutionary change with respect to chromosome number among the vertebrate lineages [10].

Various studies have investigated the molecular and evolutionary events that have yielded the highly unusual Indian muntjac karyotype. Emerging from those studies is the hypothesis that tandem chromosome fusion events occurred during the evolution of the muntjac lineage, resulting in the small number of large chromosomes seen in the modern-day Indian muntjac [12,13]. Molecular cytogenetic studies have provided the most compelling evidence for such a tandem chromosome fusion hypothesis. These have demonstrated the presence of centromeric satellite repeats [14] and telomeric repeats [15] at interstitial positions of Indian muntjac chromosomes by fluorescence *in situ *hybridization (FISH) and established Indian muntjac-human comparative genome maps by chromosome painting [16,17]. Comparative FISH studies using chromosome-specific probes derived from flow-sorted muntjac chromosomes have further suggested that the extant Indian muntjac karyotype was derived from an ancestral deer karyotype with a diploid genome of 70 chromosomes that underwent a series of chromosome fusion events and other chromosomal rearrangements [18]; several putative junction regions (that is, genomic sites where ancestral chromosomes have fused together) were identified in these studies.

More recently, four telomeric-satellite I repeat junctions on Indian muntjac chromosomes were sequenced [19,20], and mapping studies with bacterial artificial chromosomes (BACs) helped to define the orientation of other putative ancestral chromosome fusion sites in the Indian muntjac genome [21]. Specifically, evidence for centromere-telomere (head-to-tail) fusions was encountered in the arms of Indian muntjac chromosomes, whereas that for centromere-centromere (head-head) fusions was found at the centromeres. The 46-chromosome karyotype of the Chinese muntjac is thought to have evolved from a common 70-chromosome ancestral species through 12 tandem fusions involving 18 chromosomes [22]. Additional studies using cross-species BAC mapping suggested that the tandem fusions that occurred during the karyotypic evolution of the closely related species *Muntiacus crinifrons*, *M. feae*, and *M. gongshanensis *also had a centromere-telomere (head-to-tail) orientation [23,24].

The above model for muntjac chromosome evolution involves a number of known repetitive sequences that are associated with either telomeres or centromeres. For example, as with certain other mammals, muntjacs harbor the repeat (TTAGGG)_n _at their telomeres [15]. In some mammals (for example, human), this repeat unit is also found intrachromosomally in various forms: subtelomerically (including degenerate instances), as head-to-head (that is, telomere-to-telomere) fusion products [25], and within short, essentially exact repeat stretches [26]. Also implicated in the above model are Cervidae-specific centromeric satellite sequences, including satellites I, II, and IV. Satellite I (MMVsatIA in Indian muntjac [27], C5 in Chinese muntjac [14]) is roughly 1 kb in length and contains internal 31 bp subrepeats [28,29]. Satellite II (Mmv-0.7 in Indian muntjac [30]) is 0.9 kb in length. Immunoprecipitation of Indian muntjac DNA with human anti-centromere autoantibodies has shown that satellite II associates with centromeric protein A (CENPA) [31] and participates in the formation of the muntjac kinetochore. Satellite IV (MMV-1.0 in Indian muntjac, MR-1.0 in Chinese muntjac) is roughly 1 kb in length and is highly similar to satellite II [32]. Characterization of these three satellite sequences in the Formosan muntjac (*Muntiacus reevesi micrurus*), a subspecies of Chinese muntjac with the same number of chromosomes, revealed the following chromosomal organization: pter - II - IV - I - qter [33]. Additionally, satellite II (FM-satII in Formosan muntjac) co-localizes with telomeric sequences on Formosan muntjac chromosomes [33], indicating that Formosan (and likely Chinese) muntjac chromosomes are truly telocentric.

We sought to use comparative sequencing to establish a more detailed view of the evolutionary events leading to the unusual Indian muntjac karyotype. Here, we report the sequencing and characterization of a number of genomic regions in the Indian muntjac genome that represent sites of ancestral chromosome fusion events. Comparative analyses of the generated sequences with the orthologous genomic regions in the Chinese muntjac and several other mammalian species reveal details about the molecular architecture and the likely evolutionary history of the Indian muntjac genome.

## Results

### BAC isolation, mapping, and characterization

We reasoned that Indian muntjac BACs containing regions corresponding to ancestral chromosome fusion sites would likely contain remnant telomeric-repeat sequences. We thus screened an approximately 11-fold redundant Indian muntjac BAC library with probes specific for telomeric repeats, and found that a large number of clones (approximately 3,000 or approximately 1.4% of the library) yielded at least a weak hybridization signal. The 343 BACs with the strongest hybridization signals were selected for further study. Restriction enzyme digest-based fingerprint analysis [34] allowed the selected clones to be assembled in 45 multi-clone contigs. Southern blot analysis of the BACs in the 19 largest contigs (each containing at least 4 clones and averaging 6.9 clones) confirmed the presence of telomeric-repeat sequences in all clones, and these repeat sequences consistently resided on the same-sized restriction fragment in all BACs from a given contig.

**Figure 1:**
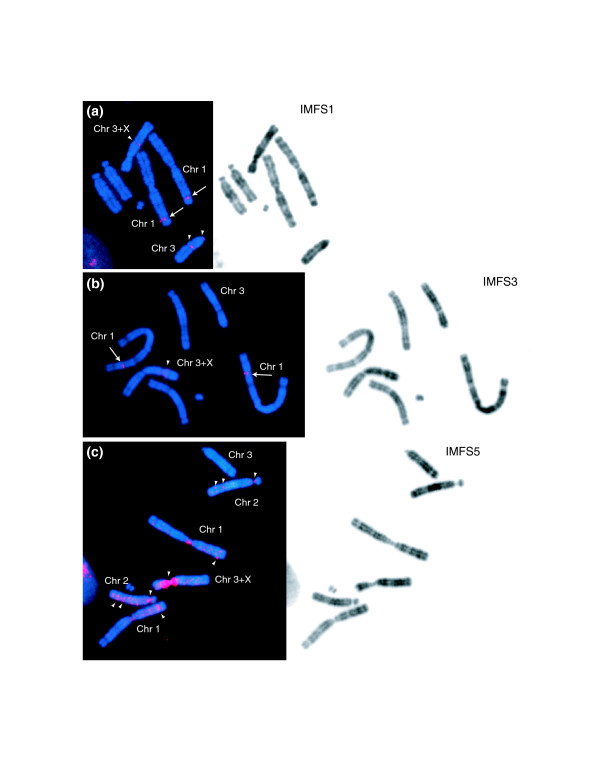
FISH mapping of Indian muntjac BACs. Three Indian muntjac BACs (whose sequences correspond to accession numbers **(a) **[GenBank:AC154146], **(b) **[GenBank:AC152355], and **(c) **[GenBank:AC154920]) were mapped by FISH to metaphase spreads prepared from an Indian muntjac fibroblast cell line. Hybridization is seen at: (a) interstitial positions on chromosomes 1 (arrows), 3, and 3+X, as well as the centromere of chromosome 3 (the signals on 3 and 3+X are indicated with arrowheads); (b) an interstitial position on chromosome 1 (arrows) and at the neck of chromosome 3+X (arrowhead); and (c) multiple sites on various Indian muntjac chromosomes (arrowheads). FISH composite images generated from merging the DAPI (blue) and Spectrum Orange (red) channels (left) and inverted DAPI banding images (right) are provided in each case. Based on further studies (see text for details), the origins of the analyzed BACs were ultimately found to be on chromosome 1 in the case of (a, b), but not yet established in the case of (c); further, the analyzed clones were found to contain the indicated ancestral chromosome fusion sites (IMFS1, IMFS3, and IMFS5, respectively; Table 1).

Southern blot analysis was also performed using probes designed from previously reported muntjac satellite-telomeric repeat junctions [19]. BACs from 7 of the 19 largest contigs were found to hybridize to at least 1 of these probes; representative BACs from each of these 7 contigs were studied further by FISH. The presence of various repeats (for example, telomeric- and centromeric-repeat sequences) in the isolated BACs typically resulted in complex FISH patterns, as illustrated Figure 1. Strong hybridization signals were observed at the euchromatin/heterochromatin boundary of the X centromere. These hybridization results are similar to those obtained with other probes containing satellite I (for example, C5 and TGS400) [14,19]. With this initial set of clones, we at best encountered BACs hybridizing strongly to only two locations in the Indian muntjac genome (Figures 1a,b); typically, the analyzed clones hybridized to many more sites (Figure 1c).

### Generation and assimilation of Indian and Chinese muntjac genomic sequences

Based on our BAC mapping and characterization studies, we selected and sequenced [35] one clone from each of the above seven Indian muntjac BAC contigs. We also sequenced a small number of Indian muntjac BACs ([GenBank:AC154147], [GenBank:AC154148], and [GenBank:AC154923]; data not shown) that appeared to contain telomeric repeats based on hybridization studies, but were not positive with any of the probes designed from previously reported muntjac satellite-telomeric repeat junctions. Subsequently, we isolated and sequenced additional overlapping BACs to extend the sequence coverage of these regions. In total, we generated the sequence for 18 BACs (Table 1), yielding approximately 1.88 Mb of Indian muntjac genomic sequence.

**Table 1:** Generated sequences of Indian muntjac chromosome fusion sites

Name	Accession numbers*	Sequence length (bp)^†^	Number of sequencing gaps^‡^	(TTAGGG)_n _repeats (bp)^§^	Total satellite I (bp)^¶^	Indian muntjac chromosome^¥^
IMFS1	[GenBank:DP000824]	292,157	9	367^#^	22,121	1
IMFS2	[GenBank:DP000825]	209,878	3	616	36,382	1
IMFS3	[GenBank:DP000827]	460,923	7	24, 25, 162, 341	105,769**	1
IMFS4	[GenBank:DP000826]	324,188	2	22, 413	12,225	3
IMFS5	[GenBank:DP000830]	172,824	2	168	72,301**	Unknown
IMFS6	[GenBank:DP000828]	248,686	2	185^#^, 261^#^	61,861	2
IMFS7	[GenBank:DP000829]	174,711^††^	2	837	87,048	Unknown

*Each IMFS sequence was assembled using the generated sequences from two or more BACs (except for IMFS5, which was derived from a single BAC sequence). The indicated GenBank accession numbers correspond to the assembled sequences (also see Figures 3 and 6). ^†^Length of the assembled IMFS sequence. ^‡^Total number of gaps within the sequences of the individual BACs used to generate the IMFS sequence. Note that there are no gaps in the regions spanning the telomeric and satellite I repeats. ^§^Total size of (TTAGGG)_n _block within the assembled IMFS sequence. In some cases, the (TTAGGG)_n _blocks are interspersed with other sequences, in which case more than one size is given. ^¶^Total amount of satellite I sequence (present in a single block except in the case of IMFS2 and IMFS4; Figure 3). ^¥^Indian muntjac chromosome localization, as determined by FISH studies of individual BACs (Table 3). ^#^A similar repeat, (TTCGGG)_n_, resides immediately adjacent to the (TTAGGG)_n _block. **Satellite I sequence resides in a single block that is interrupted by L1 and MER66-int LTR/ERV1 repeats. ^††^The chromosome fusion site within IMFS7 contains a region of 100% sequence identity with TGS400 [19].

We also sequenced six Chinese muntjac BACs (approximately 0.99 Mb total) derived from genomic regions (Chinese muntjac telomere (CMTel)1, CMTel3, CMTel4, and Chinese muntjac satellite (CMSat)4; Table 2) that are orthologous to some of the generated Indian muntjac sequence. In all cases, sequence contigs were ordered and oriented, ensuring that the correct long-range organization of the sequence was established [35]. In some cases, sequence gaps were filled using standard sequence-finishing routines [36]. Each set of overlapping BAC sequences was assembled into a single non-redundant sequence, which in turn was manually verified and submitted to GenBank ([GenBank:DP000820-DP000830]; Tables 1 and 2). Each of the resulting seven assembled Indian muntjac genomic sequences are presumed to contain a different ancestral chromosome fusion site (designated IMFS1 to IMFS7 for Indian muntjac fusion site; Table 1). A previously reported junction sequence (TGS400) [19] lies within IMFS7. The other six IMFSs, although of similar repeat composition, do not match any of the previously described junction sequences [19,20], indicating that they represent novel chromosome fusion sites.

**Table 2:** Chinese muntjac sequences orthologous to Indian muntjac chromosome fusion sites

Name	Orthologous sequence*	Accession numbers^†^	Sequence length (bp)^‡^	Number of sequencing gaps^§^	(TTAGGG)_n _repeats (bp)^¶^
CMTel1	IMFS1	[GenBank:DP000822]	202,617	4	None
CMTel3	IMFS3	[GenBank:DP000821]	292,786	3	24
CMTel4	IMFS4	[GenBank:DP000820]	286,938	3	None
CMSat4	IMFS4	[GenBank:DP000823]	215,295	5	None

*CMTel reflects Chinese muntjac sequence that is orthologous to the telomeric side of the corresponding IMFS sequence; CMSat reflects Chinese muntjac sequence that is orthologous to the satellite side of the corresponding IMFS sequence. CMTel sequences are not necessarily subtelomeric in the Chinese muntjac genome, but were likely subtelomeric in a shared ancestor with Indian muntjac. Similarly, CMSat sequences are not necessarily pericentromeric in the Chinese muntjac genome, but were likely pericentromeric in a shared ancestor with Indian muntjac (see text). The relationship between IMFS and CMTel/CMSat sequences is shown in Figures 3 and 6. Cytogenetic localization of individual BACs used to generate Chinese muntjac sequences is provided in Table 4. ^†^The indicated GenBank accession numbers correspond to each sequenced BAC used to generate the assembled Chinese muntjac sequence. ^‡^Length of the assembled multi-BAC or individual BAC Chinese muntjac sequence. ^§^Total number of gaps within the sequences of individual BACs used to generate the CMTel/CMSat sequence. ^¶^Total size of (TTAGGG)n block within the assembled sequence.

### Detection of repetitive and duplicated sequences

**Figure 2:**
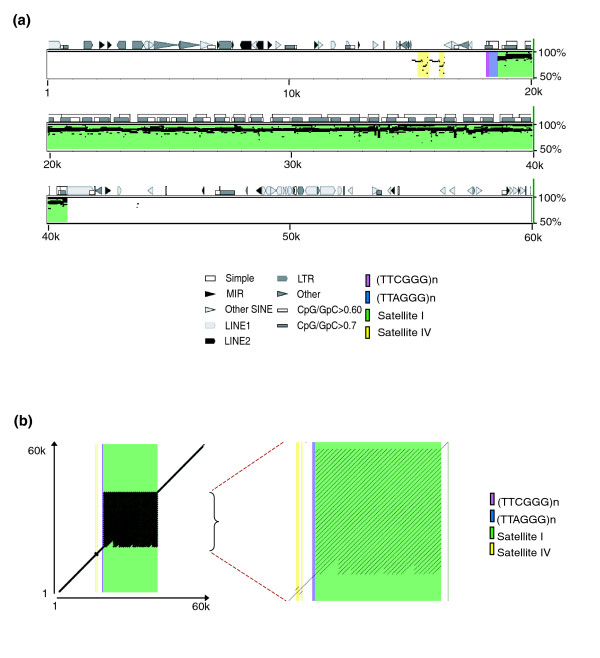
Self-self comparative sequence analysis of an Indian muntjac chromosome fusion site. A 60 kb sequence within IMFS1 was compared to itself using PipMaker [63]. **(a) **Pip plot reveals the putative chromosome fusion site, which consists of a stretch of telomeric repeats (TTAGGG)_n _(blue), and then a large segment of centromeric satellite I (green); note that the latter has extensive amounts of self-self aligning sequences (reflecting satellite I monomers with high sequence identity). Also highlighted are additional features of interest: satellite IV (yellow) and a short stretch of (TTCGGG)_n _(purple). **(b) **Dot plot of the same 60 kb region shown in (a). Expanded view reveals the periodic nature of the satellite I monomers.

**Figure 3:**
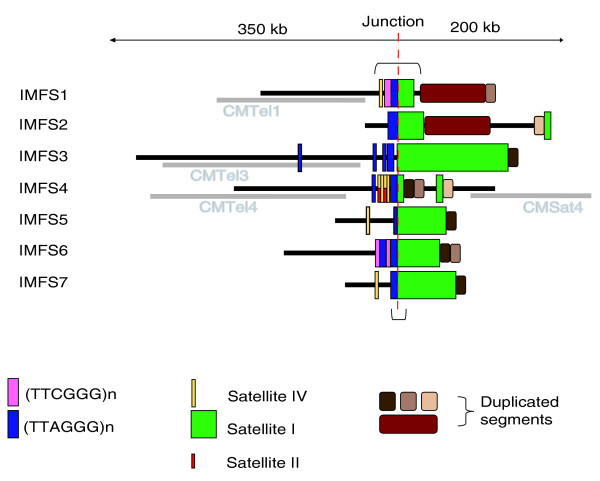
Long-range organization of chromosome fusion sites in Indian muntjac. The content and organization of the seven generated Indian muntjac sequences (black lines) is depicted. The positions of (TTAGGG)_n _(blue), (TTCGGG)_n _(purple), satellite I (green), and satellite IV (yellow) blocks as well as duplicated segments (brown and beige) are indicated. Generated orthologous Chinese muntjac sequences are shown in gray (for IMFS1, IMFS3, and IMFS4 only). The junction is defined as the point where the (TTAGGG)_n _telomeric repeats are fused with satellite I repeats (red dashed line). The bracketed area of IMFS1 indicates the region depicted in Figure 2; the bracketed area of IMFS7 indicates the region matching TGS400 [19].

Characterization of the generated Indian muntjac sequences involved detection and classification of repetitive sequences. In addition to typical transposable-element repeats (for example, long interspersed nucleotide elements (LINEs), short interspersed nucleotide elements (SINEs), and long terminal repeats (LTRs)), we paid particular attention to the presence of known telomeric and centromeric repeats (Figure 2). All seven Indian muntjac sequences listed in Table 1 (IMFS1 through IMFS7) were found to contain at least one major block of the telomeric repeat (TTAGGG)_n _(Figure 3); these blocks range from 168 to 837 bases in length. Sequences IMFS4, IMFS6, and IMFS3 have 1, 1, and 3 additional telomeric-repeat blocks, respectively; the additional blocks are shorter than the others (22 to 185 bases). In all cases, the individual (TTAGGG)_n _monomers are oriented in the same direction; in no case did we encounter a head-to-head configuration (5'(TTAGGG)_n_-(CCCTAA)_n_3'; for example, as found on human chromosome 2q13 [37]). In IMFS1 and IMFS6, a similar repeat - (TTCGGG)_n _- resides immediately adjacent to the more common (TTAGGG)_n _telomeric repeat (Figure 3).

Additionally, all seven Indian muntjac sequences were found to contain centromeric satellite I repeat sequences immediately adjacent to the telomeric-repeat block (Figure 3), similar to that found previously in the Indian muntjac genome [19]. The amount of satellite I differs among the sequences, ranging from roughly 12 kb (IMFS4) to over 105 kb (IMFS3). The satellite I sequences are rarely interrupted by other repeats; exceptions include the presence of MER66-int long terminal repeat/endogenous retrovirus (LTR/ERV) and L1 repeats that interrupt the satellite I sequences in IMFS5 and IMFS3, respectively. Of note, IMFS1, IMFS4, IMFS5, and IMFS7 also contain a small block of centromeric satellite IV sequence [32] (Figure 3). IMSF4 additionally has two short blocks of satellite II on the opposite strand of the satellite IV repeat; satellites II and IV are known to be highly similar [32].

Pericentromeric regions of mammalian chromosomes frequently harbor segments that are present in more than one copy in the genome [38]. These duplicated segments typically originate from various ancestral genomic locations and are physically juxtaposed with centromeric satellites. Copies of each duplicated segment usually have high pair-wise sequence identity due to the relatively recent occurrence of the duplication event.

We analyzed the generated Indian muntjac sequences for the presence of duplicated segments. In all 7 of the chromosome fusion sites characterized here, at least one duplicated segment was found to reside immediately adjacent to satellite I (Figure 3). Further, all duplicated segments depicted in Figure 3 are at least 1 kb in size (typically much larger) and share 94-98% pair-wise sequence identity with their matching colored block(s) in Figure 3. Of note, the duplicated segment present in IMFS2 and IMFS4 (light beige block in Figure 3) is an exception, having only 82% sequence identity between copies. IMFS1 has a large (>60 kb; reddish brown block in Figure 3) duplicated segment that is also present in IMFS2; within this duplicated segment are 5 regions that are over 3 kb in size, which share 95-98% sequence identity (3 of these regions are over 11 kb in size), and which reside in the same relative order and orientation in IMFS1 and IMFS2. An approximately 10 kb duplicated segment (brown block in Figure 3) is present in 5 other chromosome fusion sites (IMFS3-IMFS7), with 94-97% pair-wise sequence identities. IMFS1, IMFS4, and IMFS6 share another duplicated segment that is greater than 10 kb in size and has 96-98% sequence identify among copies (beige block in Figure 3). Different combinations and spatial arrangements of these duplicated segments are seen among the seven chromosome fusion sites (Figure 3).

Similar analyses were performed with the generated orthologous Chinese muntjac sequences. As with the Indian muntjac sequences, generic classes of repeats (for example, LINES, SINES, and LTRs) were identified. Additionally, a short block of (TTAGGG)_n _repeats (24 bp) was found in CMTel3; no centromeric satellites were found in any of the Chinese muntjac sequences. Consistent with the Chinese muntjac sequences being of telomeric and not centromeric origin, none contain duplicated segments (based on comparisons with each other and with their orthologous Indian muntjac sequences; Figure 3). This result was expected given that the Chinese muntjac BACs were selected to be orthologous to the non-repetitive regions of IMFSs.

### Synteny analysis and gene annotation

**Figure 4:**
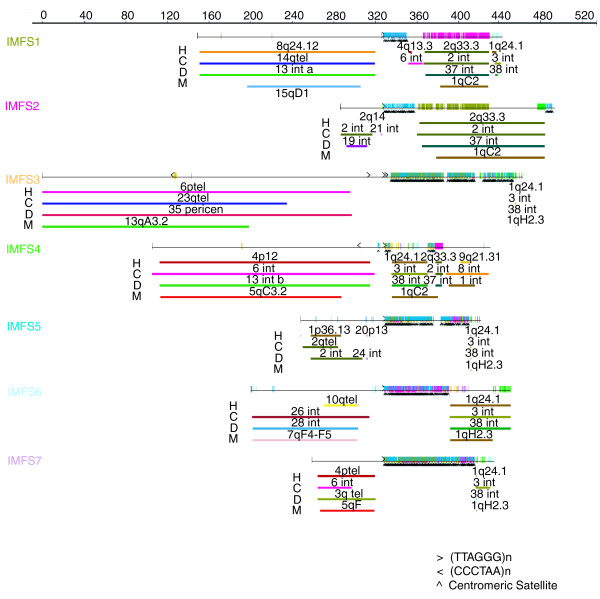
Synteny relationships between IMFS sequences and corresponding human, cow, dog, and mouse genome sequences. Each generated Indian muntjac sequence (IMFS1-IMFS7) is depicted and represented by a different color; the vertical hatch marks on each sequence indicate identity with other IMFS sequences and are colored to correspond with those IMFS sequences. Telomere (TTAGGG)_n _repeats are indicated with black arrows per their orientation; also indicated are centromeric satellite sequences (both with a black caret symbol and shaded blue). Tracks below each IMFS depict regions of synteny with the indicated genome (H, human; C, cow; D, dog; and M, mouse) as determined by BLAST-based alignments, with the chromosome location in the respective genome indicated in each case.

We performed a systematic analysis to establish the synteny relationships of the IMFS sequences relative to the human, cow, dog, and mouse genomes. For all seven IMFS sequences, the regions immediately flanking the putative ancestral chromosome fusion site were found to be orthologous to a different chromosome in all of the other species, indicating a breakage of synteny (Figure 4). For example, in the case of IMFS1: the telomeric repeat-containing side is orthologous to human chromosome 8q24.12, cow chromosome 14, dog chromosome 13, and mouse chromosome 15; and the satellite repeat-containing side contains two duplicated segments that are orthologous to human chromosomes 2q33.3 and 1q24.1, cow chromosomes 2 and 3, dog chromosomes 37 and 38, and mouse chromosome 1. There is no evidence for synteny breaks in the orthologous regions of the human, cow, dog, or mouse genomes, suggesting that the unique features of the muntjac genome are the result of relatively recent events.

Comparison of the generated Indian muntjac sequences with the human, dog, and cow genome sequences revealed the presence of a number of annotated genes (Table 3). In some instances, the duplicated segments confounded this analysis (for example, sequences matching the gene FLJ40432 reside in both IMFS1 and IMFS2, and those matching BX538248 reside in both IMFS2 and IMFS4). When available, the orthologous Chinese muntjac sequence consistently showed the same gene order and orientation as the Indian muntjac sequence (Table 4). The presence of conserved gene order within the orthologous sequences on the telomeric side of the fusion site supports the reported orthologous relationships. The juxtaposition of these gene-containing sequences with centromeric satellites and pericentromeric elements indicates a breakage of synteny relative to all of the other genomes being compared.

**Table 3:** Known genes near Indian muntjac chromosome fusion sites

Name	Telomeric side*	Function^†^	Centromeric side^‡^	Function^†^
IMFS1	Syntrophin beta1, component	Cytoskeleton	cyclin FLJ40432^¶^	Cell cycle
	*Sntb1*		AK024850^¶^	Part of frizzled 5
			Frizzled 5, Fzd5	Signal transduction
IMFS2	None	NA	cyclin FLJ40432^¶^	Cell cycle
			AK024850^5^	Part of frizzled 5
			BX538248	Unknown
IMFS3	FLJ40227^§^	Unknown	None	NA
	AK125751^§^	Unknown		
	Dual specificity protease 22, *Dsp22*	Protein tyrosine/serine/threonine phosphatase activity		
	Interferon regulatory factor 4, *Irf4*	Transcriptional activator (multiple myeloma oncogene 1)		
	Exocyst complex component 2, *Exoc2*	Transport		
IMFS4	Nuclear transcription factor, X-box binding-like, *Nfxl1*	Transcription factor	BX538248	Unknown
	Cyclic nucleotide gated channel alpha 1, *Cnga1*	Potassium ion transport		
	NIPA-like domain containing 1, *Npal1*	Unknown		
	Tyrosine kinase, *Txk*	Tyrosine protein kinase, transcription factor		
	Tyrosine-protein kinease, *Tec*	Tyrosine protein kinase, signaling		
IMFS5	None	NA	None	NA
IMFS6	ZNF cluster	Transcription factors	None	NA
	Cytochrome P450 2E1, *Cyp2e1*	Metabolism		
IMFS7	Glycosylphosphatidyl-inositol-anchor biosynthesis, *Gpi7*^¶^	Biosynthesis	None	NA

*Proximal side of telomeric-satellite I junction (Figure 3). ^†^The functionality of these genes has not been verified; table could include pseudogenes and gene fragments. ^‡^Distal side of the telomeric-satellite I junction (Figure 3), including duplicated segments. ^§^Predicted gene (UCSC Genome Browser [71]). ^¶^Provisional gene (UCSC Genome Browser). NA, not applicable.

**Table 4:** Known genes in Chinese muntjac genomic regions orthologous to Indian muntjac chromosome fusion sites

Name	Gene identity	Function*
CMTel1	Syntrophin beta1, *Sntb1*	Cytoskeleton component
CMTel3	Dual specificity protease 22, *Dsp22*	Protein tyrosine/serine/threonine phosphatase activity
	Interferon regulatory factor 4, *Irf4*	Transcriptional activator (multiple myeloma oncogene 1)
	Exocyst complex component 2, *Exoc2*	Transport
	*Hus1b*	Checkpoint protein
CMTel4	Corin, *Corin*	Serine protease
	Nuclear transcription factor, X-box binding-like, *Nfxl1*	Transcription factor
	Cyclic nucleotide gated channel alpha 1, *Cnga1*	Potassium ion transport
	NIPA-like domain containing 1, *Npal1*	Unknown
	Tyrosine kinase, *Txk*	Tyrosine protein kinase, transcription factor
	Tyrosine-protein kinease, *Tec*	Tyrosine protein kinase, signaling
CMSat4	None	NA

*The functionality of these genes has not been verified; table could include pseudogenes and gene fragments. NA, not applicable.

### Genomic locations of Indian muntjac chromosome fusion sites

**Figure 5:**
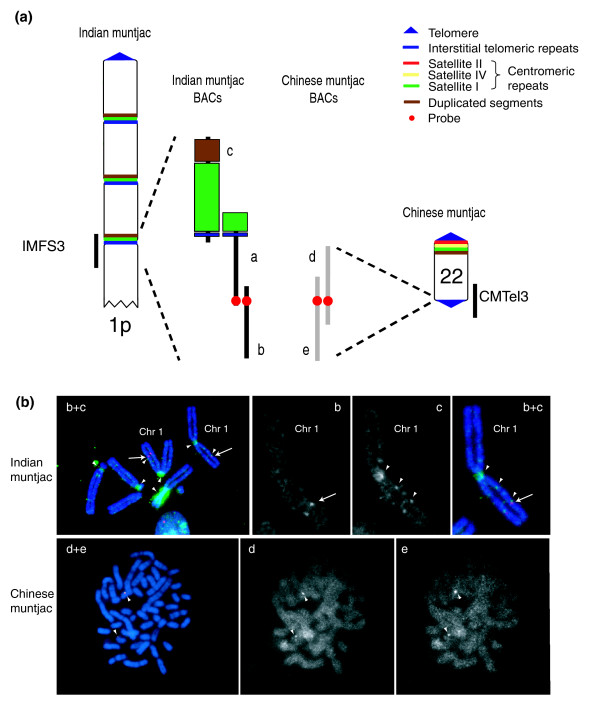
FISH-based characterization of an ancestral chromosome fusion site in the Indian muntjac genome. **(a) **Sequence IMFS3 (Table 1) was derived from three overlapping Indian muntjac BACs (BACs a-c), and contains the features expected of an ancestral chromosome fusion site (Figure 3). Each of the BACs was mapped by FISH to metaphase chromosomes from Indian muntjac cells. A probe (red circle) derived from the end of the middle clone (BAC a) was used to isolate an overlapping Indian muntjac BAC (BAC b) as well as two orthologous Chinese muntjac BACs (BACs d and e); the latter were sequenced, confirmed to be orthologous to the telomeric end of IMFS3, and designated CMTel3 (Table 2). **(b) **FISH studies were performed on the five clones (BACs a-e) depicted in (a). Indian muntjac BAC [GenBank:AC152355] (BAC a) hybridized to an interstitial position on chromosome 1 and at the neck of chromosome 3+X (shown in Figure 1b). The FISH composite image (upper row, far left) generated by merging the DAPI (blue), Spectrum Orange (red), and Spectrum Green (green) channels shows BAC [GenBank:AC197641] (BAC b; in red) hybridizing to an interstitial site on Indian muntjac chromosome 1 (arrow) and BAC [GenBank:AC166188] (BAC c; in green) hybridizing to the latter position as well as other centromeric and interstitial sites (arrowhead), reflecting its high content of satellite I and duplicated segments; an enlarged view of chromosome 1 (upper row, far right) confirms the site of co-hybridization of these two BACs. The gray scale channel images (upper row, middle) corresponding to the enlarged view of chromosome 1 show the hybridization pattern of each BAC separately. A similar FISH study with Chinese muntjac chromosomes is shown on the bottom row, with Chinese muntjac BAC [GenBank:AC196603] (BAC d; in green) and BAC [GenBank:AC198815] (BAC e; in red) co-hybridizing to a telomeric position on a pair of chromosomes (arrowheads). The two gray scale channel images show the hybridization pattern of each BAC separately. Synteny analysis suggests that the site of hybridization of these two BACs is Chinese muntjac chromosome 22 (see text).

We sought to establish the genomic locations of the generated Indian muntjac sequences and to trace their evolutionary history relative to the Chinese muntjac genome. IMFS3, the largest generated sequence (spanning >450 kb; Table 1), contains the expected features of an ancestral chromosome fusion site: a telomeric-repeat block, satellite I [14], duplicated segments, and a novel breakage of synteny (Figures 3 and 4). IMFS3 was assembled using sequences of an initial (TTAGGG)_n_-containing BAC [GenBank:AC152355] and two overlapping clones ([GenBank:AC197641] and [GenBank:AC166188]; Figure 5a). FISH studies revealed that BAC [GenBank:AC152355] mapped to Indian muntjac chromosome 1 (Figure 1b), while BACs ([GenBank:AC197641] and [GenBank:AC166188]; Figure 5b) both hybridized to a pair of interstitial sites on chromosome 1. BAC [GenBank:AC166188] also hybridized to various centromeres and other interstitial sites, likely due to the presence of satellite I and duplicated segments.

**Figure 6:**
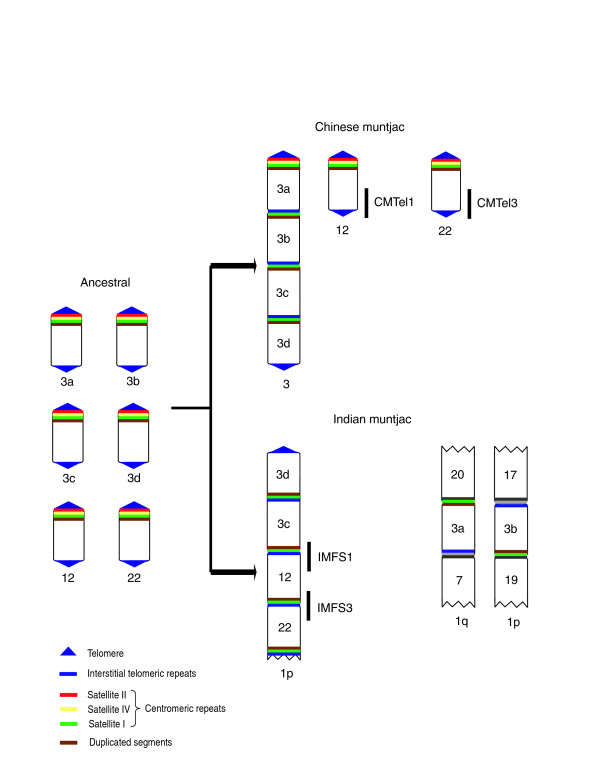
Evolutionary history of Indian muntjac chromosome fusion sites. A proposed model is shown tracing the evolutionary history of Indian muntjac IMFS1 and IMFS3 as well as the orthologous Chinese muntjac sequences CMTel1 and CMTel3. The hypothetical ancestral muntjac genome contained as many as 70 chromosomes, a small subset of which is shown on the left. There is evidence [18,39] that Chinese muntjac chromosome 3 was derived from three fusion events involving ancestral chromosomes 3a-3d. Ancestral chromosomes 3d, 3c, 12, and 22 appeared to have fused in head-to-tail fusions to form the distal end of Indian muntjac chromosome 1p; note that ancestral chromosomes 3a and 3b fused with other chromosomes and are present elsewhere in the Indian muntjac genome [18,39]. The chromosome fusion sites on Indian muntjac chromosomes contain telomeric repeats adjacent to satellite I sequences (Figures 2 and 3), consistent with sequential head-to-tail fusions of telocentric chromosomes. During these events the telomeric centromere of the 'head' chromosome (containing telomeric repeats and satellite II and IV sequences) becomes lost, and satellite I sequences become fused with telomeric repeats from the 'tail' chromosome.

We were able to generate Chinese muntjac sequence orthologous to IMFS3. Two overlapping Chinese muntjac BACs (isolated with a probe derived from Indian muntjac BAC [GenBank:AC152355]) were sequenced, resulting in contig CMTel3 (Table 2). FISH studies revealed that both clones ([GenBank:AC196603] and [GenBank:AC198815]) co-localize to a pair of Chinese muntjac telomeres. Sequences on the telomeric side of IMFS3 as well as CMTel3 sequences are orthologous to the telomeric region of human chromosome 6p25.3 and cow chromosome 23qtel. These sequences contain genes *Dusp22*, *Irf4*, and *Exoc2 *(Tables 3 and 4). Based on the known synteny relationships among the human, cow, and Chinese muntjac genomes [39], we can deduce that CMTel3 maps to chromosome 22 in the Chinese muntjac genome. Thus, IMFS3 appears to contain the fusion site between the telomere of an ancestral chromosome related to Chinese muntjac chromosome 22 and, based on the comparative chromosome map [21] and the presence of satellite I and duplicated segments, the centromere of an ancestral chromosome related to Chinese muntjac chromosome 12 (Figures 5a and 6).

In a similar fashion, we established that IMFS1 resides on Indian muntjac chromosome 1. IMFS1 includes the assembled sequence derived from the two Indian muntjac BACs, while the orthologous CMTel1 represents the sequence of the Chinese muntjac BAC. Indian muntjac BAC [GenBank:AC189002] and Chinese muntjac BAC [GenBank:AC187414] were isolated with a probe derived from Indian muntjac BAC [GenBank:AC154146] (Figure 1a). FISH studies revealed that both Indian muntjac BACs map to Indian muntjac chromosome 1, while the Chinese muntjac BAC maps to a pair of Chinese muntjac telomeres. Sequences on the telomeric side of IMFS1 as well as CMTel1 sequences are orthologous to human chromosome 8q24.14 and cow chromosome 14qtel in a genomic region that contains *Sntb1 *(Tables 3 and 4). Based on the known synteny relationships among the human, cow, and Chinese muntjac genomes [39], we can deduce that CMTel1 maps to chromosome 12 in the Chinese muntjac genome. Thus, IMFS1 appears to contain the fusion site between the telomere of an ancestral chromosome related to Chinese muntjac chromosome 12 and, based on the comparative chromosome map [21] and the presence of satellite I and duplicated segments, the centromere of an ancestral chromosome related to Chinese muntjac chromosome 3c (Figure 6).

The remaining five Indian muntjac sequences (IMF2, IMF4, IMF5, IMF6, and IMF7) could not be unambiguously matched to predicted chromosome fusion sites [21] based on the available data, as detailed below.

The sequence on the telomeric side of IMFS2 is orthologous to the telomeric region of human chromosome 2q14.3 and cow chromosome 2. Based on the known synteny relationships (see above), we can deduce that the IMFS2 telomeric side maps to Chinese muntjac chromosome 3. Using the same logic as above, IMFS2 thus appears to contain the fusion site between the telomere of an ancestral chromosome related to Chinese muntjac chromosome 3 and the centromere of another ancestral chromosome. Based on the comparative chromosome map [21] and our FISH studies of IMFS2, there are three potential matching fusion sites on Indian muntjac chromosome 1: 3c/3d, 3b/17, and 3a/20.

The sequence on the telomeric side of IMFS4 as well as CMTel4 sequences are orthologous to human chromosome 4p12 and cow chromosome 6. These sequences contain the genes *Corin*, *Nfxl1*, *Cnga1, Npal1*, *Txc*, and *Tec *(Tables 3 and 4). Based on the known synteny relationships (see above), we can deduce that CMTel4 maps to Chinese muntjac chromosome 16 or 21. Using the same logic as above, IMFS4 thus appears to contain the fusion site between the telomere of an ancestral chromosome related to Chinese muntjac chromosome 16 or 21 and the centromere of an ancestral chromosome related to Chinese muntjac chromosome 21 or 8. Based on the comparative chromosome map [21] and our FISH studies of IMFS4, there are two potential matching fusion sites on Indian muntjac chromosome 3+X: 16/21 and 21/8.

The sequence on the telomeric side of IMFS5 is orthologous to human chromosome 1p36.13 and cow chromosome 2qtel. Based on the known synteny relationships (see above), we can deduce that the IMFS5 telomeric side is also orthologous to Chinese muntjac chromosome 3. Using the same logic as above, IMFS5 thus appears to contain the fusion site between the telomere of an ancestral chromosome related to Chinese muntjac chromosome 3 and the centromere of another ancestral chromosome. Based on the comparative chromosome map [21] and our FISH studies of IMFS5, there are three potential matching fusion sites on Indian muntjac chromosome 1: 3c/3d, 3b/17, and 3a/20.

We established that IMFS6 resides on Indian muntjac chromosome 2. The sequence on the telomeric side of IMFS6 is orthologous to human chromosome 10q26.3 and cow chromosome 26 (in a genomic region that contains *Cyp2e1*; Table 3). Based on the known synteny relationships (see above), we can deduce that the IMFS6 telomeric side is also orthologous to Chinese muntjac chromosome 2. Using the same logic as above, IMFS6 thus appears to contain the fusion site between the telomere of an ancestral chromosome related to Chinese muntjac chromosome 2 and the centromere of another ancestral chromosome. Based on the comparative chromosome map [21] and our FISH studies of IMFS6, there are four potential matching fusion sites on Indian muntjac chromosome 2: 2b/2c, 2c/2d, 2d/2a, and 2a/10.

Finally, the sequence on the telomeric side of IMFS7 is orthologous to human chromosome 4ptel and cow chromosome 6 (in a genomic region that contains *Gpi7*; Table 3). Based on the known synteny relationships (see above), we can deduce that the IMFS7 telomeric side is also orthologous to Chinese chromosome 16 or 21. Using the same logic as above, IMFS7 thus appears to contain the fusion site between the telomere of an ancestral chromosome related to Chinese muntjac chromosome 16 or 21 and the centromere of an ancestral chromosome related to Chinese muntjac chromosome 21 or 8. Based on the comparative chromosome map [21] and our FISH studies of IMFS7, there are two potential matching fusion sites on Indian muntjac chromosome 3+X: 16/21 and 21/8.

## Discussion

The strikingly small diploid chromosome number in the Indian muntjac has captured the interest of geneticists for a number of years [2]. The rarity of such a karyotype among mammals suggests that the extant Indian muntjac genome formed through an unusual set of evolutionary events. Here, we applied the tools of comparative genomics to gain clues about that evolutionary history.

Using a BAC-based mapping and sequencing strategy, we isolated, sequenced, and analyzed seven regions of the Indian muntjac genome that appear to reflect ancestral chromosome fusion sites. These genomic regions share a similar organization, containing both specific repeats (telomeric and satellite I) in the expected spatial configuration [19] and duplicated segments (Figures 2 and 3). The architecture of the telomeric side of each ancestral chromosome fusion site is generally less complex than that of the satellite I side. There is clear evidence for breakage of synteny at each site, with the orthology (relative to four other mammalian genomes) on the telomeric side interrupted precisely at the fusion site itself (Figure 4). For the telomeric side of several sites, we further generated orthologous Chinese muntjac sequence, and demonstrated that these sequences emanate from authentic telomeric regions of Chinese muntjac chromosomes (Figure 5).

Our findings provide new details about the chromosome fusion events that led to the Indian muntjac karyotype. With the exception of IMFS7 (which corresponds to TGS400 [19]), the IMFS sequences reported here represent novel, previously unreported Indian muntjac chromosome fusion sites [19,20]. Like previously reported fusion sites (TGS400, TGM225, and TCS165 [19]), the IMFS sequences contain juxtaposed satellite I and telomeric repeats. In all cases, immediately adjacent to satellite I are duplicated genomic segments, a feature that is typical of mammalian pericentromeric regions (Figure 3). This architecture contrasts the observed order of satellite sequences within either the telomeres or centromeres of muntjac chromosomes. For example, in the Formosan muntjac, the order of satellite sequences at the telocentric end of chromosomes is pter - II - IV - I - qter [33]. If this order of centromeric satellites reflects that present in the common ancestral species, the formation of head-to-tail chromosome fusions likely proceeded with a loss of most of the telomeric sequence from the 'tail' chromosome and a loss of telomeric, satellite II, and satellite IV (and potentially some satellite I) sequences from the 'head' chromosome. This would have created new physical associations between pericentromeric duplicated segments and satellite I sequences from the donor 'head' chromosome and telomeric sequences from the donor 'tail' chromosome (Figures 3 and 6). Together with the reported general reduction in intron sizes in Indian muntjac [20], the loss of most of the telomeric repeats and satellites II and IV could at least partially account for the smaller genome of Indian (haploid C-value of 2.1 pg) versus Chinese (haploid C-values of 2.7) muntjac [7].

Muntjac satellite II is functionally important, having been shown to bind CENPA. The complex of satellite DNA and CENPA marks the site of the active centromere [31]. Multi-centric chromosomes can form multiple spindle attachments and have been shown to be lost during cell division. [40-42]. It is thus interesting to note that the loss of satellite II (associated with the chromosome fusion events that occurred during the evolution of the muntjac genome) likely ensured that there were not multiple active centromeres in the resulting Indian muntjac chromosomes.

The main telomeric-repeat block as well as the smaller (TTAGGG)_n _blocks (in IMFS3, IMFS4, and IMFS6) and (TTAGGG)_n_-like blocks (in IMFS1 and IMFS6; Figure 3) represent additional evidence that these regions reflect ancestral telomeres, as such sequences are often found in subtelomeric regions. The presence of small blocks of satellite IV (in IMFS1, IMFS4, IMFS5, and IMFS7) and satellite II (in IMFS4; Figure 3) within the telomeric side of some of these sites suggests that these centromeric satellites may have been present near the telomeres of ancestral muntjac chromosomes (or could also suggest that a more complex genomic rearrangement occurred during the chromosome fusion event).

To our knowledge, the current study is the first to reveal the presence of duplicated genomic segments on the satellite I side of muntjac chromosome fusion sites. A duplicated segment orthologous to human chromosome 1q24.1 is present in all of the IMFS sequences reported here except IMFS2 (Figure 4), suggesting that this may have been a commonly duplicated segment within pericentromeric regions of ancestral muntjac chromosomes. Human and rodent pericentromeric regions are known to contain various segmental duplications [38,43,44]. Of note, the duplicated segments characterized here (Figures 3 and 4) might be useful as molecular landmarks for identifying additional IMFS sequences in the Indian muntjac genome.

Previous studies have suggested that chromosome fusions are produced by sequence-specific recognition and illegitimate recombination between homologous DNA elements (or other specific motifs) on non-homologous ancestral chromosomes [14,15,18,19,22,30]. The repetitive elements identified in the seven IMFS sequences (telomeric repeats, duplicated segments, and satellites I, II, and IV) represent potential candidate targets for such illegitimate recombination events. Exploring the presence and organization of these repetitive elements at orthologous locations in the Chinese muntjac genome would provide additional insights about their involvement in the chromosome fusion events leading to Indian muntjac karyotype.

The general model for chromosome fusion events that involves telomeric repeats and satellite I is supported by the data reported here (Figures 3 and 6), but does not necessarily account for all of the chromosome fusion sites in the Indian muntjac genome. Many more Indian muntjac fusion sites remain to be isolated and characterized; indeed, 29 tandem, head-to-tail fusions would theoretically be required to condense the estimated 70 ancestral chromosomes into the current set of Indian muntjac chromosomes [21]. These additional chromosome fusion sites may differ structurally from the seven described here. Further, a different type of chromosome fusion event (head-to-head, centric fusion) is likely to have yielded the current centromeres of the Indian muntjac chromosomes [12,13,21]. Meanwhile, the Chinese muntjac karyotype appears to have been derived from a common ancestral species through 12 tandem fusions involving 18 chromosomes [22]. Sequencing and characterizing additional chromosome fusion sites in both the Indian and Chinese muntjac genomes would enable a more complete delineation of the evolutionary history of these various fusion events.

It is notable that the chromosome fusion sites we identified in Indian muntjac are architecturally different to the characterized fusion site on human chromosome 2q13 [37] and other regions in the human genome containing interstitial telomeric repeats [26]. The Indian muntjac chromosome fusion sites contain telomeric repeats immediately adjacent to satellite I [19], suggesting that these repetitive sequences played a role in the fusion event. In contrast, most interstitial telomeric sequences in the human genome appear to have been derived from double-strand breakage and repair via non-homologous end-joining rather than from telomeric fusion events [25]. We isolated and characterized interstitial telomeric sequences in an additional three Indian muntjac BACs ([GenBank:AC154147], [GenBank:AC154148], and [GenBank:AC154923]; data not shown); these clones contain what appears to be short interstitial telomeric sequences similar to those described in humans and other organisms [25,26]. For example, [GenBank:AC154147] has a short block of telomeric repeats flanked by mammalian-wide interspersed repeats/short interspersed elements (MIR/SINE) that resembles the human class B interstitial telomeric sequences described previously [26]. Note that these BAC sequences were excluded from the main studies reported here because they did not contain closely linked telomeric repeats and satellite I nor was there any evidence of synteny breakage; thus, they are unlikely to represent chromosome fusion sites.

## Conclusion

Our studies help to provide a better understanding of the likely evolutionary history of the Indian muntjac karyotype as well as insights into the paradoxical finding that closely related species can harbor genomes with drastically different organizations. More broadly, the comparative studies of genomes, such as that performed here, are providing insights into how mammalian genomes evolve, why mammals most typically package their genomes into 40-60 chromosomes, and how unusually large (or small) mammalian chromosomes replicate and segregate.

## Materials and methods

### BAC isolation

BAC clones were isolated from libraries constructed from Indian (CHORI-244) and Chinese (CHORI-245) muntjac [45]; each library was derived from a male individual. The initial isolation of Indian muntjac BACs involved the use of end-labeled telomere repeat-specific oligonucleotide probes [(TTAGGG)_5 _and (CCCTAA)_5_]. Subsequent isolation of Indian and Chinese muntjac BACs involved the use of 'overgo' probes, which consist of 36-bp double-stranded, radiolabeled DNA molecules generated by performing primer extension with two 22mer oligonucleotides that contain an 8-base complementary region of overlap at their 3' ends [46]; overgo probes were designed from the ends of sequenced BACs (see below; probe sequences available on request).

### BAC contig assembly

Following isolation, BACs were subjected to restriction enzyme digest-based fingerprint analysis [34], allowing their assembly in clone contigs [47,48]. In some cases, contigs were extended by isolating overlapping clones using new overgo probes designed from the ends of sequenced BACs.

### BAC characterization

BAC clones were cultured shaking in 2X YT medium (Quality Biological, Gaithersburg, MD, USA) containing 12.5 μg/ml chloramphenicol at 37°C overnight, and BAC DNA was purified using an Autogen 740 Automated Plasmid Isolation System (Holliston, MA, USA). For Southern blot analysis, the purified DNA was digested with *Eco*RI, electrophoretically separated in a 1% agarose gel, and transferred to Hybond-N membranes (GE Healthcare Bio-sciences, Piscataway, NJ, USA) using standard procedures [49]; membranes were then hybridized, and the resulting images were captured with an FLA-5000 phosphorimager (Fujifilm). Hybridization probes used to analyze the Southern blots included: (TTAGGG)_5 _and (CCCTAA)_5 _telomeric probes (as above); and modified (longer) overgo probes designed from known muntjac satellite-telomeric repeat junctions reported in Hartmann and Scherthan [19]: AGGGTTAGGGTGGAGGCCGCAAATTCAACCTCCCTC and GAGGCTTCTCGCAGCTGTAGGTCTGGTTGAGGGAGG from TGS400 [GenBank:AY322158]; AGGGTTAGGGCATCTCGGGGTCGATTCCAGTGGAGG and ATCTGGGAGAGGGACGTTGAATTTATGGCCTCCACT from TGM225 [GenBank:AY322159]; and ACCCTAACCCTTTGACTGTGTGGATGAAAAGGGGCA and CCCCTGCACTGCGTGCAGAGAAATTCCGTGCCCCTT from TCS165 [GenBank:AY322160]. For FISH studies, the purified BAC DNA was labeled with Spectrum Orange or Spectrum Green d-UTP (Abbott Laboratories, Des Plaines, IL, USA), and hybridized to Indian and Chinese muntjac metaphase spreads [50-52].

### Culturing muntjac cell lines

A fibroblast cell line from a male Indian muntjac (AG15826) was obtained from the Coriell Cell Repositories and cultured in EMEM medium (BioWhittaker, Walkersville, MD, USA) supplemented with 10% fetal bovine serum, 1% glutamine, 1% penicillin-streptomycin, 2× vitamins (Invitrogen, Carlsbad, CA, USA), and 2× amino acids (Sigma-Aldrich, St. Louis, MO, USA). A fibroblast cell line from a male Chinese muntjac was kindly provided by Dr BR Brinkley (Baylor College of Medicine) and cultured in Opti-MEM I medium supplemented with 4% fetal bovine serum, 1% glutamine, and 1% penicillin-streptomycin (Invitrogen).

### Generation and analysis of muntjac-BAC sequences

Selected BACs were sequenced as part of the NISC Comparative Sequencing Program [53], as described [48]. Following refinement [35], high-quality sequences of overlapping BACs were compiled into a single non-redundant sequence using the program TPF Processor [54]. Repetitive sequences were identified and classified with RepeatMasker (version open-3.1.6) [55] using a cow repeat library (Repbase Update 20061006, RM database version 20061006) [56,57].

Known muntjac satellite sequences were identified with NCBI BLAST 2 Sequences [58,59] or RepeatMasker using a custom repeat library compiled for each muntjac species. The Indian muntjac repeat library contained Indian muntjac satellites I [GenBank:X02323], II [GenBank:AF170123], and IV [GenBank:AY064466]. The Chinese muntjac repeat library contained Chinese muntjac satellites I [GenBank:X56823] and IV [GenBank:AY064467] as well as Formosan muntjac satellite II [GenBank:AY380828]. Sequences were annotated for known genes based on matches to human [60-62] (or cow [63], when available) and RefSeq mRNA sequences using Spidey [64]. Multi-species sequence comparisons were performed using MultiPipMaker [65]. Duplicated genomic segments were detected and analyzed using PipMaker [66] and/or LAGAN/VISTA [67].

### Synteny analysis

The generated and assembled Indian muntjac sequences were first analyzed by RepeatMasker (version open-3.1.6) using the cow repeat database (Repbase Update 20061006, RM database version 20061006). The coordinates of satellite and (TTAGGG)_n _tracts were identified, and all sequences matching cow repeats were masked. Each repeat-masked sequence was then used to query (using BLASTN) a database consisting of all Indian muntjac repeat-masked sequences, and non-self matches with >90% identity and >50 bp in length (reflecting duplicated segments among the sequences) were identified. Similarly, each repeat-masked sequence was used to query (using BLASTN) the assembled genome sequences of human (hg18, NCBI build 36.1) [60-62], cow (Btau_4.0) [63], dog (canFam2) [68], and mouse (mm9, build 37) [69] that had been downloaded from the UCSC Genome Browser [70]. Human, dog, and mouse sequence matches with a bit score of >100 and cow sequence matches with a bit score of >600 were displayed. The regions of synteny to the human, cow, dog, and mouse genome-sequence assemblies are shown in Figure 4.

## Abbreviations

BAC: bacterial artificial chromosome; CENPA: centromeric protein A; CMSat: Chinese muntjac satellite; CMTel: Chinese muntjac telomere; FISH: fluorescence *in situ *hybridization; IMFS: Indian muntjac fusion site; LINE: long interspersed nucleotide element; LTR: long terminal repeat; SINEs: short interspersed nucleotide elements.

## Authors' contributions

VT carried out the laboratory-based studies, performed the primary bioinformatics analyses, and drafted the manuscript. MGS advised on numerous aspects of the study, reviewed the results of the bioinformatics analyses, and performed critical reading and editing of the manuscript. SH and HR performed the synteny analysis and contributed text and a figure to the manuscript. The NISC Comparative Sequencing Program generated the genomic sequence data. AD and EP performed the FISH studies. EDG conceived of the study, participated in its design and coordination, and performed critical editing of all components of the manuscript. All authors read and approved the final manuscript.
